# Identifying Membrane Protein Types Based on Lifelong Learning With Dynamically Scalable Networks

**DOI:** 10.3389/fgene.2021.834488

**Published:** 2022-03-14

**Authors:** Weizhong Lu, Jiawei Shen, Yu Zhang, Hongjie Wu, Yuqing Qian, Xiaoyi Chen, Qiming Fu

**Affiliations:** ^1^ School of Electronic and Information Engineering, Suzhou University of Science and Technology, Suzhou, China; ^2^ Suzhou Key Laboratory of Virtual Reality Intelligent Interaction and Application Technology, Suzhou University of Science and Technology, Suzhou, China; ^3^ Provincial Key Laboratory for Computer Information Processing Technology, Soochow University, Suzhou, China; ^4^ Suzhou Industrial Park Institute of Services Outsourcing, Suzhou, China

**Keywords:** lifelong learning, membrane proteins, dynamically scalable networks, position specific scoring matrix, evolutionary features

## Abstract

Membrane proteins are an essential part of the body’s ability to maintain normal life activities. Further research into membrane proteins, which are present in all aspects of life science research, will help to advance the development of cells and drugs. The current methods for predicting proteins are usually based on machine learning, but further improvements in prediction effectiveness and accuracy are needed. In this paper, we propose a dynamic deep network architecture based on lifelong learning in order to use computers to classify membrane proteins more effectively. The model extends the application area of lifelong learning and provides new ideas for multiple classification problems in bioinformatics. To demonstrate the performance of our model, we conducted experiments on top of two datasets and compared them with other classification methods. The results show that our model achieves high accuracy (95.3 and 93.5%) on benchmark datasets and is more effective compared to other methods.

## 1 Introduction

The biological cell’s daily activities are associated with membranes, without which it would not be possible to form a living structure. The essential proteins that make up membranes are the lipids and proteins that are the main components of membranes. In the present biological research, there are eight types of membrane proteins: 1) single-span 1; 2) single-span 2; 3) single-span 3; 4) single-span 4; 5) multi-span; 6) lipid-anchor; 7) GPI-anchor and (8) peripheral (Cedano et al., 1997).

Bioinformatics is present in all aspects of the biological sciences, and how to use computers to classify proteins efficiently and accurately has been a hot research problem in the direction of bioinformatics and computer science. Although traditional physicochemical as well as biological experiments are desirable in terms of predictive accuracy, these methods are too cumbersome and require a great deal of human and material resources. To save time and financial costs, and to better understand the structure and function of membrane proteins, a number of calculations have been developed to efficiently discriminate between protein types (Feng and Zhang, 2000; Cai et al., 2004; Zou et al., 2013; Wei et al., 2017; Zhou et al., 2021; Zou et al., 2020; Qian et al., 2021; Ding et al., 2021; Ding et al., 2021; Zou et al., 2021). The extant methods are in large part improvements on Chou’s algorithm (Chou and Elrod, 1999). Song et al. (Lu et al., 2020) used Chou’s 5-step method to extract evolutionary information to input to a support vector machine for protein prediction. Cao and Lu (Lu et al., 2021) avoided loss of information due to truncation by introducing a fag vector and used a variable length dynamic two-way gated cyclic unit model to predict protein. Yang (Wu et al., 2019) designed a reward function to model the protein input under full-state reinforcement learning. Wu and Huang (Wu et al., 2019) et al. used random forests to build their own model and used binary reordering to make their predictions more efficient. To avoid the limitations of overfitting, Lu and Tang (Lu et al., 2019) et al. used an energy filter to make the sequence length follow the model adaptively.

In most methods of machine learning, predictions are made using fixed models for different kinds of proteins, which generally suffer from two problems. Firstly, they do not allow for incremental learning, and secondly, they do not consider task-to-task connections at the task level. Lifelong learning approaches aim to bridge these two issues. Lifelong machine learning methods were first proposed by Thrun and Mitchell (Thrun and Mitchell, 1995), who viewed each task as a binary problem to classify all tasks. A number of memory-based and neural network-based approaches to lifelong learning were then proposed and refined by Silver (Silver, 1996; Silver and Mercer, 2002; Silver et al., 2015) et al. Ruvolo and Eaton (Ruvolo and Eaton, 2013) proposed the Efficient Lifelong Learning Algorithm (ELLA), which greatly enhanced the algorithm proposed by Kumar (Kumar and Daume, 2012) et al. for multi-task learning (MTL). Ruvolo and Eaton (Ruvolo and Eaton, 2013) viewed lifelong learning as a real-time task selection process, Chen (Chen et al., 2015) proposed a lifelong learning algorithm based on plain Bayesian classification, and Shu (Shu et al., 2017) et al. investigated the direction of lifelong learning by improving the conditional random field model. Mazumder (Chen and Liu, 2014) investigated human-machine conversational machines and enabled chatbots to learn new knowledge in the process of chatting with humans. Chen, Liu and Wang (Wang et al., 2016) proposed a number of lifelong topic modeling methods to mine topics from historical tasks and apply them to new topic discovery. Shu et al. proposed a relaxed labeling approach to solve lifelong unsupervised learning tasks. Chen and Liu (Chen and Liu, 2019) provide more detailed information on the direction of lifelong machine learning in this book.

**FIGURE 1:**
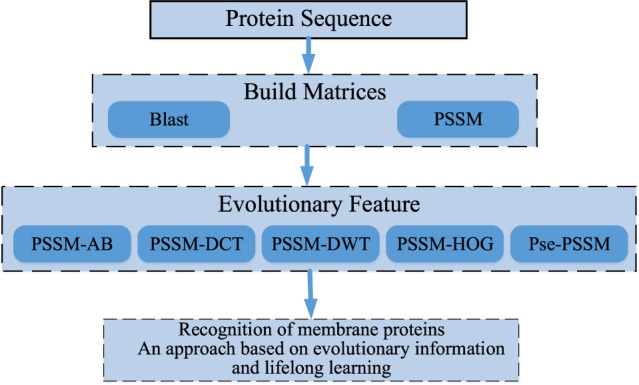
The summary of the main research.

After a lot of research and careful selection, we ended up using a DSN model based on sequence information and lifetime learning of membrane proteins themselves. First, we processed the membrane protein sequence dataset based on BLAST (Altschul et al., 1997) to obtain the scoring matrix (PSSM) (Dehzangi et al., 2017; Sharma et al., 2018; Yosvany et al., 2018; Chandra et al., 2019). Then, we extracted valid features from the PSSM by the averaging block method (Avblock) (Shen et al., 2019), the discrete wavelet transforms method (DWT) (Shen et al., 2017; Wang et al., 2017), the discrete cosine transforms method (DCT) (Ahmed et al., 1974), the histogram of oriented gradients method (HOG) (Qian et al., 2021)and the Pse-PSSM method. The features extracted by these five methods are then stitched end-to-end and fed into our model for prediction. Finally, the performance is evaluated by random validation tests and independent tests. Through the results we can see that our model achieves good prediction results in the case of predicting membrane protein types. Figure 1 shows a sketch of the main research in this paper.

## 2 Materials and Methods

**FIGURE 2:**
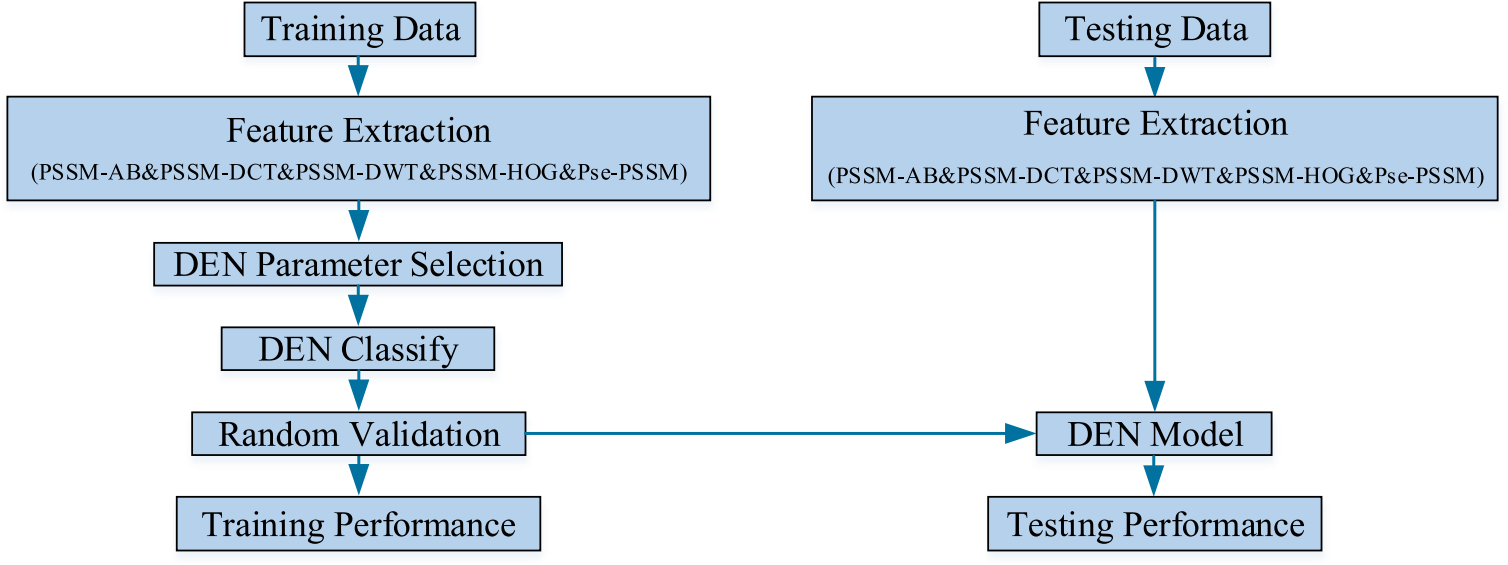
The research infrastructure for this approach.

In this experiment, the sequence of discriminating membrane protein types can be broadly divided into three steps: creating the required model, performing training and testing of the model, and making predictions and conducting analysis of the results. Firstly, the features are extracted from the processed dataset. Next, the features are integrated into a lifelong learning model for prediction. Finally, the sequence information of the membrane proteins is transformed into algorithmic information, which is then analyzed and predicted using the model. Figure 2 shows the research infrastructure for this approach.

### 2.1 Analysis of Data Sets

In order to test the performance of our lifelong learning model, we experimentally selected two membrane protein datasets for testing, Data 1 and Data 2. The specifics of the two datasets are shown in Table 1. Data 1 and Data 2 include eight membrane protein types. Data 1 is from the work of Chou (Chou and Shen, 2007) et al. The training and test sets were randomly obtained from Swiss-Prot (Boeckmann et al., 2003) by percentage assignment, which ensured that the quantities of these two sequences are consistent. Data 2 is from the work of Chen (Chen and Li, 2013) et al. where they used the CD-hit (Li and Godzik, 2006) method to remove redundant sequences from dataset 1 so that no two-by-two sequences would have less than 40% identity.

**TABLE 1:** The sample sizes for the two different data sets used in this experiment.

The specific type of classification	Dataset 1		Dataset 2	
	Train	Test	Train	Test
Single-span type 1	610	444	388	223
Single-span type 2	312	78	218	39
Single-span type 3	24	6	19	6
Single-span type 4	44	12	35	10
Multi-span type 5	1,316	3,265	936	1,673
Lipid-anchor type 6	151	38	98	26
GPI-anchor type 7	182	46	122	24
Peripheral type 8	610	444	472	305
Totality	3,249	4,333	2,288	2,306

### 2.2 Extracting the Message of Evolutionary Conservatism

The PSSM used in this experiment is the “Position-Specific Scoring Matrix.” This scoring matrix stores the sequence information of membrane proteins. We use the PSSM matrix (Shen et al., 2017; Ding et al., 2017; Shen et al., 2019) for membrane protein prediction because it reflects the evolutionary information of membrane proteins very well. For any membrane protein sequence, such as Q, the PSSM can be derived by PSI-BLAST (Altschul et al., 1997), after several iterations. First it forms the PSSM based on the first search result, then it performs the next step which is the second search based on the first search result, then it continues with the second search result for another time and repeats this process until the target is searched for the best result. As the performance of the experimental results is best after three iterations, we generally adopt three iterations as the setting. The value of its E is 0.001. Assume that the sequence Q = $q_{1}q_{2}q_{3}...\;.\;q_{L}$ , whose length is L. This is followed by storing the PSSM containing the membrane protein evolution information inside a matrix with a size-area of L **×** 20. The matrix is represented as follows:

$$\mathrm{PSS}M_{\mathrm{original}}={\left[\begin{array}{cc} \begin{array}{cc} p_{\mathrm{1,1}} & p_{\mathrm{1,2}}\\ p_{\mathrm{2,1}} & p_{\mathrm{2,2}}\\ \vdots & \vdots \end{array} & \begin{array}{cc} \ldots & p_{\mathrm{1,20}}\\ \ldots & p_{\mathrm{2,20}}\\ \ddots & \vdots \end{array}\\ \begin{array}{cc} p_{i,1} & p_{i,2}\\ \vdots & \vdots \\ p_{L,1} & p_{L,2} \end{array} & \begin{array}{cc} \ldots & p_{i,\mathrm{20}}\\ \ddots & \vdots \\ \ldots & p_{L,\mathrm{20}} \end{array} \end{array}\right]}_{\mathrm{L\times}\mathrm{20}}$$ (1)

In addition, the expression below shows the representation of $\;PSSM_{original}\left(i,j\right)$ :

$$\mathrm{PSS}M_{\mathrm{original}}\left(\mathrm{i,j}\right)=\overset{\mathrm{20}}{\underset{\mathrm{k=1}}{\sum }}\omega \left(\mathrm{i,k}\right)\mathrm{\times D}\left(\mathrm{k,j}\right)\mathrm{, i=1, \ldots ,L}\mathrm{. j=1,\ldots ,20}$$ (2)

where ω(i,k) is the frequencies of the k-th type of amino acid at the i-th position and D(k,j) is the mutation percentage of the substitution matrix from the k-th molecular substances into the sequence of the protein. The larger the value, the more conserved the position is. If this is not present, the contrary will be achieved.

#### 2.2.1 Pse-Pssm

Pse-PSSM (pseudo-PSSM) is a feature extraction method often used in membrane protein prediction (Chou and Shen, 2007). PSSM matrices are often used in the characterization of membrane proteins. This feature extraction method, which aims to preserve PSSM biological information through pseudo amino acids, is expressed as follows:

$$f_{\mathrm{i,j}}=\frac{p_{\mathrm{i,j}}-\frac{1}{\mathrm{20}}\sum_{\mathrm{k=1}}^{\mathrm{20}}p_{\mathrm{i,k}}}{\sqrt{\frac{1}{\mathrm{20}}\sum_{\mathrm{l=1}}^{\mathrm{20}}{\left(p_{\mathrm{i,l}}-\frac{1}{\mathrm{20}}\sum_{\mathrm{k=1}}^{\mathrm{20}}p_{\mathrm{i,k}}\right)}^{2}}}\mathrm{,i=1,\ldots ,L;j=1,\ldots ,20}$$ (3)

The $P_{normalized}$ is as follows:

$$P_{\mathrm{normalized}}={\left[\begin{array}{ccc} f_{\mathrm{1,1}} & \ldots & f_{\mathrm{1,20}}\\ \begin{array}{c} \vdots \\ \begin{array}{c} f_{i,1}\\ \vdots \end{array} \end{array} & \begin{array}{c} \ddots \\ \begin{array}{c} \ldots \\ \ddots \end{array} \end{array} & \begin{array}{c} \vdots \\ \begin{array}{c} f_{i,\mathrm{20}}\\ \vdots \end{array} \end{array}\\ f_{L,1} & \ldots & f_{L,\mathrm{20}} \end{array}\right]}_{L\times \mathrm{20}}$$ (4)

where $f_{i,j}\;$ is the normalised PSSM score with a mean of 0 for the 20 amino acids. And the $p_{i,j}$ is the raw score. While a positive score refers to the occurrence of the corresponding homozygous mutation, which is more frequent in multiple reciprocals, over and above chance mutations, a negative score is the opposite of a positive score.

#### 2.2.2 Average Blocks

The AB method was first proposed by Huang et al. Its full name is the averaging block methodology (AvBlock) (Jong Cheol Jeong et al., 2011). When feature extraction is performed for PSSM, the extracted feature values are diverse because the size of individual features is different and the abundance of amino acids also varies in the individual membrane proteins. To solve this type of problem, we can average the features for the local features of the PSSMs. Inside each module after averaging, 5% of the membrane protein sequences are covered, and this method is the AB feature extraction method. When performing the AB method feature extraction on the PSSM, it is not necessary to consider the sequence length of the membrane proteins. When we split the PSSM matrix by rows, it becomes a block of size L/20 each, with 20 blocks. After this operation, every 20 features form a block. the AB formulation is as follows:

$$\mathrm{AB}\left(k\right)=\frac{\mathrm{20}}{N}\overset{\frac{N}{\mathrm{20}}}{\underset{\mathrm{p=1}}{\sum }}\mathrm{Mt}\left(\mathrm{p+}\left(\mathrm{i-}1\right)\times \frac{\mathrm{20}}{N}\mathrm{,j}\right),\mathrm{i=}\mathrm{1,}\mathrm{\ldots ,20;}\mathrm{j=}\mathrm{1\ldots ,20;}j\mathrm{=1,\ldots ,20;}\mathrm{k=j+}\mathrm{20\times}\mathrm{(i-}\mathrm{1)}$$ (5)

Where N/20 is the size of j blocks and $Mt\left(p+\left(i-1\right)\times \frac{20}{N},j\right)$ is a vector of size 1 × 20 from position $i_{th}$ of the $j_{th}$ block that is taken in the pssms.

#### 2.2.3 Discrete Wavelet Transform

We refer to a discrete wavelet transform feature extraction method as DWT, which uses the concepts of frequency and position (Nanni et al., 2012). It is because we can consider the membrane protein sequence as a picture, then matrix the sequence and extract the coefficient information from the matrix by DWT. This method was first suggested by Nanni et al.

**FIGURE 3:**
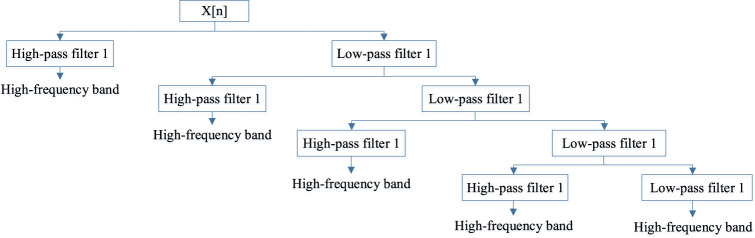
The 4-stage DWT structure.

In addition to this, we refer to the projection of the signal f(t) onto the wavelet function as the wavelet transform (WT). This is shown below:

$$T\left(\mathrm{a, b}\right)=\frac{1}{\sqrt{a}}\overset{t}{\underset{0}{\int }}\mathrm{f(t)\psi}\left(\frac{\mathrm{t-b}}{a}\right)d_{t}$$ (6)

where in the above equation, the scale variable is denoted by a, the translational variable by b, and $\psi \left(\frac{t-b}{a}\right)$ refers to the wavelet parsing and analysis function. T (a, b) refers to the transform coefficients used in conjunction with a particular location when performing a specific wavelet period signal transform. Further, an efficient DWT algorithm was submitted by Nanni et al.; they denote the discrete signal f(t) by x [n] and perform a DWT on it. It is expressed in terms of the coefficients as follows:

$$y_{\mathrm{j,low}}\left[n\right]=\overset{N}{\underset{\mathrm{k=1}}{\sum }}x\left[k\right]g\left[2\mathrm{n-k}\right]$$ (7)

$$y_{\mathrm{j,high}}\left[n\right]=\overset{N}{\underset{\mathrm{k=1}}{\sum }}x\left[k\right]h\left[2\mathrm{n-k}\right]$$ (8)

where N is the length of the discrete signal and the low-pass and high-pass filters are g and h, respectively. $y_{j,low}\left[n\right]$ is the approximate coefficient when the signal is in the low-frequency part, while $y_{j,high}\left[n\right]$ is the detailed coefficient when the signal is in the high-frequency band. In our study, their mean, standard deviation, maximum and minimum values are computed through the DWT quadruple layer. In addition, the PSSM discrete signal after transforming four times, consists of 20 discrete signals. The 4-stage DWT structure can be seen in Figure 3.

#### 2.2.4 Discrete Cosine Transform

The DCT (Ahmed et al., 1974), known as the Discrete Cosine Transform, converts a signal to its fundamental frequency by means of a linearly separable transform. This method has been widely used in the field of image compression. In this experiment, we compressed the PSSM matrix of membrane proteins using a 2-dimensional DCT (2D-DCT). 2D-DCT is defined as follows:

$$F_{\mathrm{PSSM-DCT}}=\alpha_{i}\alpha_{j}\overset{\mathrm{M-}1}{\underset{\mathrm{m=}0}{\sum }}\overset{\mathrm{N-}1}{\underset{\mathrm{n=}0}{\sum }}\mathrm{PSSM(m,n)cos}\frac{\pi \left(\mathrm{2m+}1\right)i}{\mathrm{2M}}\cos\frac{\pi \left(\mathrm{2n+}1\right)j}{\mathrm{2N}}$$ (9)

$$\alpha_{i}\{\begin{array}{c} \sqrt{\frac{1}{M}}\mathrm{,i=}0\\ \sqrt{\frac{2}{M}},1\mathrm{\leq i\leq M-}1 \end{array}\alpha_{j}\{\begin{array}{c} \sqrt{\frac{1}{N}}\mathrm{,j=}0\\ \sqrt{\frac{2}{N}},1\mathrm{\leq j\leq N-}1 \end{array}$$ (10)

The mission of the DCT is to convert a uniformly distributed information density into an uneven distribution. Once its length and signal have been converted, the most important part of the information is collected in the low frequency section of the PSSM, that is in the middle and top-left corner.

#### 2.2.5 Histogram of Oriented Gradient

Histogram of Oriented Gradients (HOG), which is a method for describing features, is mainly used in computer vision. In this experiment, to handle a PSSM matrix using the HOG method, it is first necessary to look at it as a particular image matrix. In the first step, the horizontal gradient values and vertical gradient values of the PSSM are used to derive the direction and size of the gradient matrix. In the second step, the gradient matrix is divided into 25 sub-matrices by direction and size. In the third step, conversion of the results generated in the second step is carried out according to the requirement to generate 10 histogram channels per sub-matrix.

### 2.3 Lifelong Learning

**FIGURE 4:**
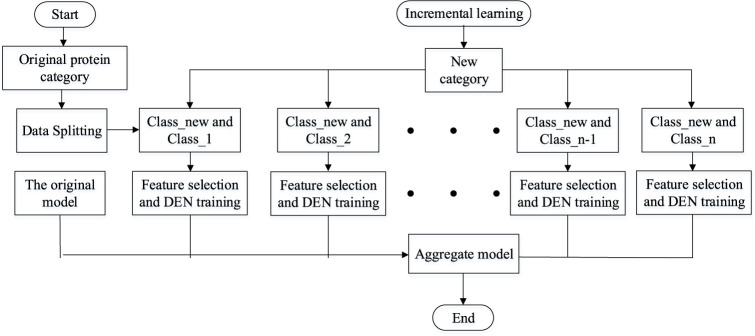
The simple flow of the lifelong learning model.

Lifelong learning (Thrun and Mitchell, 1995), like machine learning, can be divided into the directions of lifelong supervised learning, lifelong unsupervised learning and lifelong reinforcement learning. The lifelong machine learning part of the study focuses on whether the model can be extended when new categories of categories are added to the model. When the current model has been classified into n categories, if a new class of data is added, the model can somehow be adaptively expanded to classify n + 1 category. Multi-task learning models and lifelong machine learning are easily translatable to each other if we have all the original data. Whenever a new category is added to the original category and needs to be classified, only one new category needs to be added and then all the training data can be trained again to expand the new category. One obvious disadvantage of this strategy is that it wastes a lot of computational time to compute each new class, and if too many new classes are added, it may lead to changes in the model architecture for multi-task learning. This model therefore uses a dynamically scalable network to better perform incremental learning of the added tasks. Assume that the current model has successfully classified Class 1, Class 2, ... , Class *n*. When the new data class Class-new is added, the model does not need to train all the data from scratch, but only needs to expand the overall model by adding n new binary classification models. The simple flow of the lifelong learning model is shown in Figure 4.

### 2.4 Dynamically Scalable Networks

Dynamically scalable networks are incremental training of deep neural networks for lifelong learning, for which there will be an unknown amount and unknown distribution of data to be trained fed into our model in turn. To expand on this, there are now T a sequence of task learning models, t = 1, .... , t, .... , T is unbounded T in which the tasks at time point t carry training data $D_{t}={\left\{x_{i},y_{i}\right\}}_{i=1}^{N_{t}}$ . It is important to note that each subtask can be a single or a group of tasks. For simplicity, even though our approach is general for any kind of task, we only consider the two-classification problem. That is, input features $x\;\in \;R^{d}$ of y ∈ {0, 1} . One challenge with lifelong learning is that at the current time t, all previous training datasets are unavailable (if any, only from previous model parameters). The lifelong learning agent learns the model parameters $W^{t}$ by solving the following problem in a reasonable amount of time t:

$$\mathrm{minimiz}e_{W^{t}}L\left(W^{t};W^{\mathrm{t-}1},D_{t}\right)\mathrm{+ \lambda}\Omega \left(W^{t}\;\right)\mathrm{, t = 1}1\mathrm{, \ldots}$$ (11)

where L is task specific loss function, $W^{t}$ is the parameter for task t, and Ω ( $W^{t}$ ) is the regularization (e.g. element-wise $\ell_{2}$ norm) to enforce our model $W^{t}$ appropriately. In case of a neural network which is our primary interest, $W^{t}={\left\{W_{l}\right\}}_{l=1}^{L}$ is the weight tensor.

**FIGURE 5:**
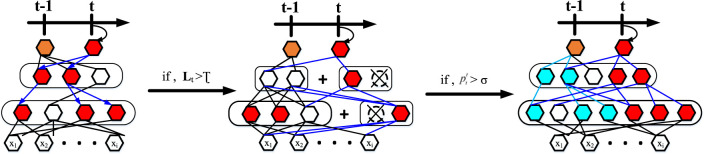
Incremental learning for dynamically scalable networks.

To counter these problems that arise in the course of lifelong learning, we allow the knowledge generated in previous tasks to be used to the maximum extent possible. At the same time, it is allowed to dynamically extend its capabilities when mechanically accumulated knowledge does not explain well for emerging tasks. Figure 5 and Algorithm 1 illustrate our progressive learning process.

## 3 Experiment Results

In this subsection we will have an analysis of the capabilities of the respective modelling and methodological approaches. Furthermore, the modelling we used in that context was compared with other available methods on separate datasets.

### 3.1 Assessment Measurements

To evaluate our lifelong learning model better, we chose several parameters: sample all-prediction accuracy (ACC), single sample specificity (SP), single sample sensitivity (SN), and Mathews correlation coefficient. These metrics are widely used in the analysis of biological sequence information:

$$\{\begin{array}{c} \mathrm{SN=}\frac{\mathrm{TP}}{\mathrm{TP+FN}}\\ \mathrm{SP=}\frac{\mathrm{TN}}{\mathrm{TN+FP}}\\ \mathrm{ACC=}\frac{\mathrm{TP+TN}}{\mathrm{TP+FP+TN+FN}}\\ \mathrm{MCC=}\frac{\mathrm{TP\times TN-FP\times FN}}{\sqrt{\left(\mathrm{TP+FN}\right)\times \left(\mathrm{TN+FP}\right)\times \left(\mathrm{TP+FP}\right)\times \left(\mathrm{TN+FN}\right)}} \end{array}\;$$ (12)

For the above equation, true trueness (TP) refers to the number of true samples correctly predicted; false positivity (FP) refers to the number of true samples incorrectly predicted; true negativity (TN) refers to the number of negative samples correctly predicted; and false negativity (FN) refers to the number of negative samples erroneously predicted (Chou et al., 2011a; Chou et al., 2011b; Chou, 2013).

### 3.2 Situational Analysis of Two Data Sets

**FIGURE 6:**
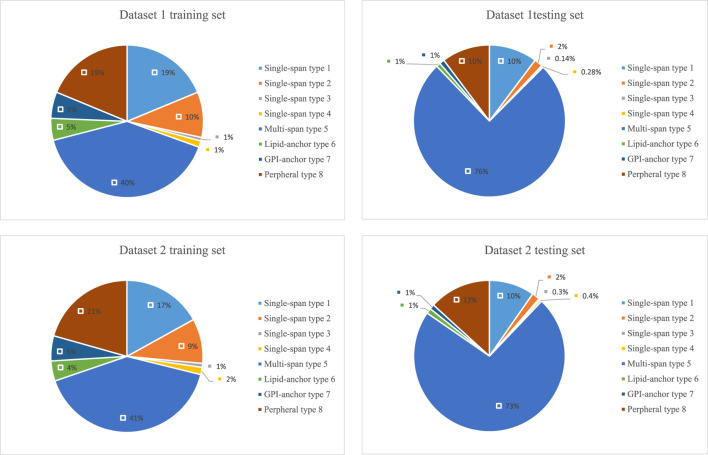
Distribution of the lengths of the training and test sets in the two datasets.

**FIGURE 7:**
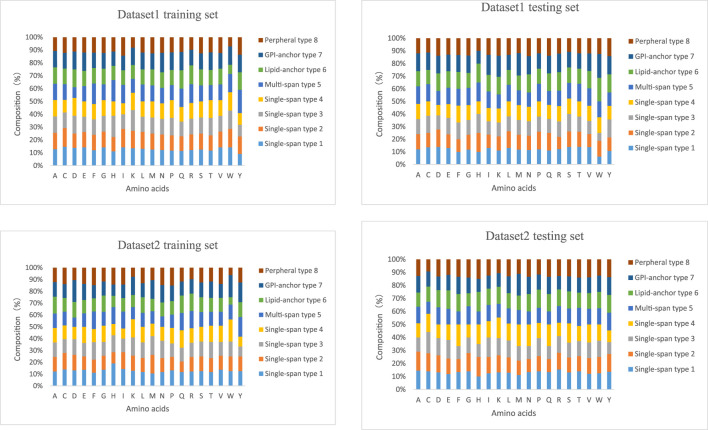
Component composition of the training and test sets of the two datasets.

The lengths of the datasets used in our experiments are shown in Figure 6. Most of the membrane proteins in dataset 1 and dataset 2 have a similar length distribution because of their specific type. To better demonstrate the superiority of lifelong learning for membrane protein classification, we calculated the amino acid frequencies for all protein types in the experiment, as shown in Figure 7.

### 3.3 The Forecasting Results for Dataset 1

The PSSM sequence matrix contains important genetic information required for protein prediction. Many elements of biological evolution, such as the stability of the three-dimensional structure and the aggregation of proteins, can have an impact on the storage and alteration of sequences. These elements demonstrate that PSSM captures important information about ligand binding. Thus, proving the validity of the PSSM characterization method.

We will compare the model methods we used in this use with other existing methods in terms of prediction accuracy on dataset 1. The methods involved in the comparison are MemType-2L (Chou and Shen, 2007) and predMPT (Chen and Li, 2013). Details can be found in Table 2, where it is clear that our model approach has an overall ACC of 95.3%. 3.7% higher than MemType-2L’s 91.6%, 2.7% higher than predMPT’s 92.6%, and 2.4% higher than Average weights’ 92.7%. In the independent test set, our method was superior for membrane protein type 2 (88.4%), type 4 (83.3%), type 5 (96.3%) and type 7 (100%).

**TABLE 2:** The performance exhibited by different models on dataset 1.

The specific type of classification	*Ave-WT* ^a^ (%)	*Mem Type-2L* ^b^ (%)	*preMPT* ^c^ (%)	*Our method*
Single-span type 1	93.9 (417/444)	86.9 (386/444)	94.6 (420/444)	94.8 (421/444)
Single-span type 2	87.2 (68/78)	70.5 (55/78)	79.5 (62/78)	88.4 (69/78)
Single-span type 3	0 (0/6)	33.3 (2/6)	33.3 (2/6)	33.3 (2/6)
Single-span type 4	66.7 (8/12)	66.7 (8/12)	41.7 (5/12)	83.3 (10/12)
Multi-span type 5	93.9 (3,065/3,265)	95.0 (3,103/3,265)	94.9 (3,097/3,265)	96.3 (3,147/3,265)
Lipid-anchor type 6	29.0 (11/38)	42.1 (16/38)	65.8 (25/38)	52.6 (20/38)
GPI-anchor type 7	84.8 (39/46)	76.1 (35/46)	93.5 (43/46)	100.0 (46/46)
Peripheral type 8	91.9 (408/444)	82.2 (365/444)	81.1 (360/444)	93.2 (414/444)
Overall	92.7 (4,016/4,333)	91.6 (3,970/4,333)	92.6 (4,014/4,333)	95.3 (4,129/4,333)

a Mean-weighted MKSVM, based.

b The results are taken from (Chou and Shen, 2007).

c The results are taken from (Chen and Li, 2013).

### 3.4 The Forecasting Results for Dataset 2

As a solution to the possible problem of untimely updates in Data 1, Chen and Li (Chen and Li, 2013) used the Swissprot annotation method to update Data Set 1, resulting in a new dataset of membrane proteins (Data Set 2). The results of the comparison using Dataset 2 are presented in Table 3. The overall average accuracy of our models was 3.2% higher than the predMPT method (90.3%). Even though they added features such as 3D structure to the predMPT prediction session, our model performance was clearly higher than it. We outperformed it by 2.6% in terms of prediction accuracy for type 1 (94.1 vs. 91.5%). In contrast, analog 5 outperformed it by 1.3% (94.1 vs. 92.8%).

**ALGORITHM 1:** Incremental Learning for Dynamically Scalable Networks.

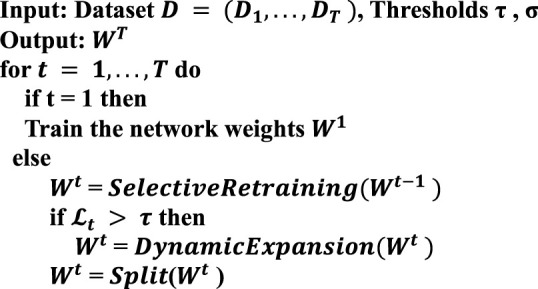

**TABLE 3:** The performance exhibited by different models on dataset 2.

The specific type of classification	*Ave-WT* ^a^ (%)	*Mem Type-2L* ^b^ (%)	*preMPT* ^c^ (%)	*Our method*
Single-span type 1	89.2 (199/223)	76.7 (171/223)	91.5 (204/223)	94.1 (210/223)
Single-span type 2	79.5 (31/39)	66.7 (26/39)	74.4 (29/39)	87.2 (34/39)
Single-span type 3	33.3 (2/6)	33.3 (2/6)	16.7 (1/6)	50.0 (3/6)
Single-span type 4	90.0 (9/10)	70.0 (7/10)	80.0 (8/10)	90.0 (9/10)
Multi-span type 5	91.1 (1,524/1,673)	91.4 (1,529/1,673)	92.8 (1,552/1,673)	94.1 (1,575/1,673)
Lipid-anchor type 6	30.8 (8/26)	23.1 (6/26)	53.8 (14/26)	57.6 (15/26)
GPI-anchor type 7	91.7 (22/26)	70.8 (17/24)	95.8 (23/24)	100.0 (24/24)
Peripheral type 8	88.9 (271/305)	68.2 (208/305)	82.6 (252/305)	93.7 (286/305)
Overall	89.6 (2066/2,306)	85.3 (1966/2,306)	90.3 (2083/2,306)	93.5 (2,156/2,306)

a Mean-weighted MKSVM, based.

b The results are taken from (Chou and Shen, 2007).

c The results are taken from (Chen and Li, 2013).

## 4 Conclusion and Discussion

In previous work, investigators have often used the PseAAC (Chou, 2001) approach to identify membrane protein types, and this approach has indeed performed well in the field of protein classification. Using Chou’s operation (Chou and Shen, 2007) on feature extraction from PSSM, we were inspired to use the five methods of Pse-pssm, DCT, AvBlock, HOG and DWT to extract features. In order to avoid the low accuracy of a single feature extraction method, we integrated the above five methods together and fed the integrated features into our DSN model method.

Our constructed lifelong learning dynamic network model proved to achieve superior results on different datasets (95.3 and 93.5%). However, the prediction of some small sample affiliations by the methodology has not been as accurate as we had anticipated. In order to improve the performance of this model, we will consider improving our own features and combining some other feature extraction methods, and adjusting the parameters of our model in our future research.

## Data Availability

Publicly available datasets were analyzed in this study. This data can be found here: https://github.com/sjw-cmd/DATA.git.
